# Metabolite Profile of Sheep Serum With High or Low Average Daily Gain

**DOI:** 10.3389/fvets.2021.662536

**Published:** 2021-05-05

**Authors:** Tao Feng, Hongxiang Ding, Jing Wang, Wei Xu, Yan Liu, Ákos Kenéz

**Affiliations:** ^1^Institute of Animal Husbandry and Veterinary Medicine (IAHVM), Beijing Academy of Agriculture and Forestry Sciences (BAAFS), Beijing, China; ^2^Joint Laboratory of Animal Science Between IAHVM of BAAFS and Division of Agricultural Science and Natural Resource of Oklahoma State University, Beijing, China; ^3^College of Animal Science and Technology, Henan University of Science and Technology, Luoyang, China; ^4^College of Animal Science and Technology, Hebei North University, Zhangjiakou, China; ^5^Department of Infectious Diseases and Public Health, City University of Hong Kong, Hong Kong, China

**Keywords:** metabolomics, sheep, average daily gain, metabolic pathway, serum

## Introduction

Sheep industry is a major branch of animal husbandry throughout the north and central parts of China mainly for mutton and wool production (1, 2). Body weight gain during the fattening period is an important determinant for carcass weight. Sheep average daily gain (ADG) refers to the average weight gain of sheep during a certain period and is an important economic growth trait that improves production efficiency and economic benefits. Specifically, ADG has been reported to have a positive correlation coefficient (*r* = 0.53) with final body weight in growing lambs (3). The ADG of lamb or sheep is affected by genetic basis, nutrition level, growth stage, and management system (4–6). In sheep feedlots, lambs with the same genetic basis, same age, same management system, and very similar initial body weight often develop a large standard deviation in ADG, accounting for unwanted differences in final body weights (7, 8). To our knowledge, few studies reported the underlying metabolic mechanism of such an inter-individual difference.

Selecting those lambs expected to have high ADG, at the earliest as possible in their longevity, or at least before the fattening period, could obviously increase the profitability of sheep feedlots (9, 10). Recently, several serum components (hormones, metabolites, hematological, and biochemical parameters) have been identified as biomarkers to evaluate the residual feed intake of sheep (11, 12), an indicator of feed conversion efficiency. Metabolomics can qualitatively and quantitatively analyze hundreds of metabolites in diverse samples, which can be extensively used to study physiological and pathophysiological process such as starving and intrauterine growth restriction in sheep (13, 14). Blood is considered as an ideal sample in sheep metabolomics research and is potentially used to reflect the metabolic status on a whole body level (15). As we have known, alterations in the blood metabolome profiles of sheep during the fattening period, particularly in fattening sheep with the same genetic basis, same age, same management system, but high or low ADG, are still unclear. Therefore, the aims of the present study were (i) to reveal the metabolic characteristics of lambs with high or low ADG under the same management system and (ii) to investigate the potential metabolic pathways related to the growth performance of sheep.

## Methods

Experiments were performed at the Experimental Station of Beijing Academy of Agriculture and Forestry Sciences in the Yangyuan county, Zhangjiakou city, Heibei province, northeast of China. A total of 200 crossbreed male lambs (*Ovis aries*) of Dorper rams and Mongolia ewes after weaning (45 days of age) were housed in eight sheltered outdoor paddocks and fed total mixed ration (TMR). Clean water and mineralized salt licks were available *ad libitum*. From 75 days of age, 50 lambs with similar body weight were selected and reared in individual pens indoors (0.7 × 1.0 m) until 120 days of age. Briefly, the lambs were acclimatized, lasting 15 days before formal assessment. At 90 days, 40 lambs with similar body weight were selected to do ADG research lasting 30 days. Lamb TMR was compounded based on the recommendations of sheep feeding standard in China (NY/T816-2004) and contained digestible energy of 11.83 MJ·kg^−1^, metabolic energy of 9.73 MJ·kg^−1^, 14.61% crude protein, 0.39% calcium, and 0.25% phosphorus. Body weight of lambs was accurately measured in the morning before feeding, and at 75, 90, and 120 days of age, using calibrated electronic scales. ADG was calculated based on body weight. Differences in ADG between the high ADG and low ADG lamb group were analyzed using a *t*-test. A *P* < 0.05 indicated statistical significance.

At 120 days of age, after weighting, blood samples were drawn from the jugular vein of the top seven lambs with the greatest ADG and the bottom seven lambs with the lowest ADG using needles and vacutainers covered with anti-coagulant (BD Vacutainer, USA) for a minimum of 6 ml. The blood was placed at room temperature for 4 h and then centrifuged at 2,000 g for 30 min at 4°C. Serum separation was carefully proceeded. The serum was aliquoted and rapidly frozen by dry ice. Frozen serum samples were stored at −80°C until metabolomics analyses.

Using 100 μl of serum, metabolites were extracted using methanol. Extracts were sonicated, and after centrifugation, the supernatant was gently added to sample vials for LC-MS/MS analysis. A pooled quality control sample (QC) was performed for system conditioning and quality control. Chromatographic separation of the metabolites was operated on a Thermo UHPLC system equipped with an ACQUITY UPLC HSS T3 (100 mm × 2.1 mm i.d., 1.8 μm; Waters, Milford, USA).

Following LC-MS/MS analyses, the raw data were inputted into the Progenesis QI 2.3 (Non-linear Dynamics, Waters, USA) for peak picking and alignment. Mass spectra of these metabolic characteristics were discerned through the accurate mass, MS/MS fragments spectra, and isotope ratio difference, by scanning in public available biochemical databases such as the Human Metabolome Database (HMDB) (http://www.hmdb.ca/) and the Metlin database (https://metlin.scripps.edu/). A multivariate statistical analysis was conducted using “ropls” (Version 1.6.2, http://bioconductor.org/packages/release/bioc/html/ropls.html) R package from Bioconductor on Majorbio Cloud Platform (https://cloud.majorbio.com). Principal component analysis (PCA) was applied to check outliers and present trends. Partial least squares discriminate analysis (PLS-DA) was used to identify the general metabolic changes in serum of sheep between high and low ADG. Variable importance in the projection (VIP) was computed by an orthogonal partial least squares discriminate analysis (OPLS-DA) model. Differential metabolites among ESI groups were summarized and annotated into their biochemical pathways through metabolic enrichment and pathway analysis based on database matching (KEGG, http://www.genome.jp/kegg/). Furthermore, Volcano plot was used to compare the size of the fold change to statistical significance.

## Results

At the beginning of the lamb fattening trial, when lambs were 90 days of age, the mean (SD in parentheses) body weight of seven original lambs corresponding to the high ADG was 26.7 (0.9) kg, while the mean body weight of seven original lambs corresponding to the low ADG was 26.8 (0.8) kg. At the end of the lamb trial, the body weight of the seven lambs with the highest weight gain was 35.67 (0.8) kg and the body weight of the seven lambs with the lower weight gain was 32.56 (1.0) kg. The ADG of high weight gain lambs was 298.1 (15.9) g·day^−1^, which differed (*P* < 0.01) from the ADG of low weight gain lambs 191.9 (23.6) g·day^−1^, while the average ADG of all lambs tested was 239.5 (34.8) g·day^−1^ (*n* = 40).

Variation of ADG depends on sheep breeds and ages. Previous studies showed that crossbreed of specialized mutton breeds and local sheep breeds had greater ADG than local sheep. For local sheep breeds under a barn feeding fattening system, 6-month Altay and Hu lambs presented ADG from 100 to 200 g·day^−1^ (16), while ADG of 3-month Ningxia Tan sheep lambs was between 90 and 130 g·day^−1^ (17) and ADG of Small Tail Han sheep lambs was between 140 and 180 g·day^−1^ (18). Furthermore, the ADG of crossbreed lambs of Dorper and Small Tailed Han sheep was between 265 and 322 g·day^−1^ in pens (19). In our study, crossbreed lambs of Dorper rams and Mongolia ewes at 4-month age exhibited excellent growth performance with an average ADG of 240 g·day^−1^. Crossbreed lambs are recommended to produce lamb meat in the north part of China.

**Figure 1:**
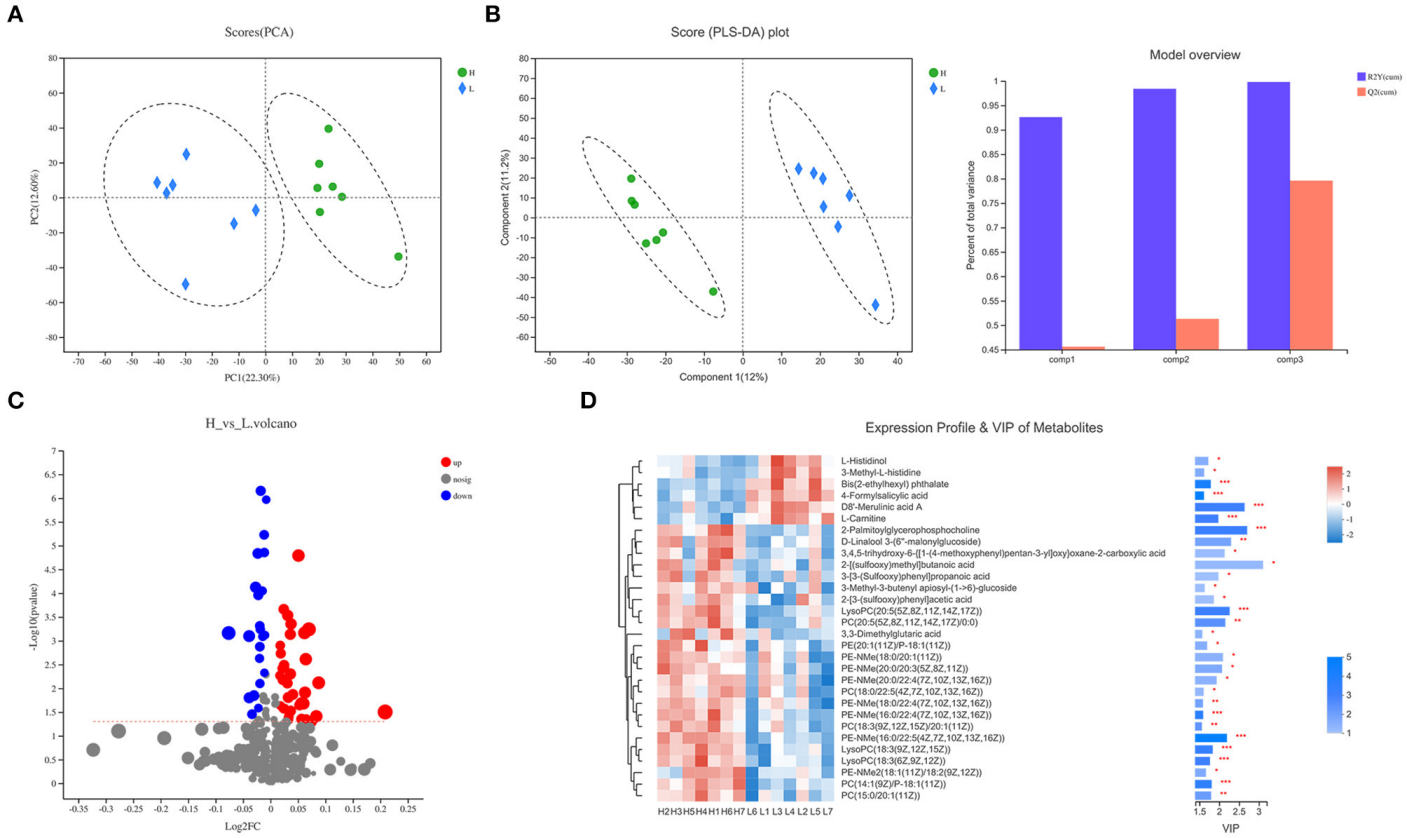
Serum metabolite profile of lambs with different average daily gain (ADG). **(A)** PCA score plot for serum metabolites of high ADG lambs (H) and low ADG lambs (L). **(B)** PLS-DA score plot for serum metabolites of high ADG lambs (H) and low ADG lambs (L), and model overview showing high R2Y and Q2. **(C)** Enhanced Volcano plots of OPLS-DA showing fold changes (log_2_FC) and the negative logarithm (base 10) of the *P*-values of 57 differential serum metabolites in high ADG lambs compared with low ADG lambs. **(D)** Expression profile, VIP score, and *P-*value of the top 30 differential serum metabolites in high ADG lambs compared with low ADG lambs.

In the LC-MS spectra of lamb serum with high or low ADG, 10,231 metabolites were initially found. After quality control and discernment, 462 compounds were reliably detected. The PCA score plot presented that the first and second principal components (PCs) clarified 22.3 and 12.6% of the variation, respectively (Figure 1A). As expected, the separated plot representing high and low ADG can be observed in the PCA plot. Next, PLS-DA was executed to exhibit the variations between the high and low ADG lambs. As shown in Figure 1B, the PLS-DA analysis demonstrated that the serum metabolites of the low ADG lambs distinctly differed from those of the high ADG lambs. Correspondingly, the values of R2Y and Q2 were 0.998 and 0.796, respectively (Figure 1), indicating good interpretability and predictability by this PLS-DA model. A value of Q2 = 1 indicates a perfect discrimination of metabolites profiles between groups.

In comparison, a total 57 differential serum metabolites were found according to the Volcano plot (*P* < 0.05, VIP > 1.0, and log_2_FC > 1 or < 1.0), of which 35 metabolites showed up-regulation and 22 showed down-regulation (Figure 1C and Supplementary Table 1). A total of 50 differential serum metabolites were annotated in six superclasses according to the HMDB database, of which 36 belonged to lipids and lipid-like molecules, 5 belonged to organic nitrogen compounds, 4 belonged to organic acids and derivatives, 2 belonged to benzenoids, 2 belonged to organic oxygen compounds, and 1 belonged to organoheterocyclic compounds. Expression profile and VIP of the top 30 metabolites based on the OPLS-DA model are shown in Figure 1D. Regarding the KEGG pathway, the four pathways including at least two differential serum metabolites annotated were metabolic pathways [L-Histidinol, Myristic acid, D-Sedoheptulose 7-phosphate, L-Arginine, and PC(14:1(9Z)/20:2(11Z,14Z))], biosynthesis of amino acids (L-Histidinol, D-Sedoheptulose 7-phosphate, and L-Arginine), glycerophospholipid metabolism [PC(14:1(9Z)/20:2(11Z,14Z)), LysoPC(20:5(5Z,8Z,11Z,14Z,17Z)), and LysoPC(18:3(6Z,9Z,12Z))], and histidine metabolism (L-Histidinol and 3-Methyl-L-histidine), respectively. Based on our results, the serum metabolome prolife of lambs was affected by high or low ADG.

As shown in Figure 1D and Supplementary Table 1, most of the lipids and lipid-like molecules were accumulated in serum samples of high ADG lambs compared with low ADG ones, including LysoPC(20:5 (5Z,8Z,11Z,14Z,17Z)) and PC(14:1(9Z)/20:2 (11Z,14Z)), which are metabolites involved in glycerophospholipid metabolism. In detail, LysoPC(20:5(5Z,8Z,11Z,14Z,17Z)) and PC(14:1(9Z)/20:2(11Z,14Z)) had a high concentration in serum of high ADG lambs [log_2_(FC) = 1.04 and 1.02]. Lysophospholipids (LPL) mainly include lysophosphatidylcholine (LPC), lysophosphatidic acid (LPA), lysophosphatidylethanolamine (LPE), and lysophosphatidylinositol (LPI), which are derivatives of phospholipid with absence of a fatty acid chain by hydrolysis (20). LPL could be a potent feed additive to improve production and feed efficiency according to studies in non-ruminant animals (21, 22), as well as in ruminants (23, 24). Recently, research showed that LPL supplementation could increase ADG in lambs, potentially through altering feed digestion (25), and intermediating bacterial phospholipid turnover as one of the cellular growth factor or potent lipid mediator in bacteria (26). Moreover, LPC could alter enterocyte monolayer permeability via protein kinase C (27).

Amino acids in tissue and in serum seemed to change under various physiological status, such as starvation, fasting, grazing, and stress in sheep (14) and dairy cows (28). During starvation in sheep, circulating amino acids had a general trend to increase mainly because muscle proteins were mobilized to improve gluconeogenesis in the livers by enhancing amino acids supply (14). Three metabolites (L-Arginine, L-Histidinol, and D-Sedoheptulose 7-phosphate) were identified to increase in circulation of low ADG lambs (Supplementary Table 1). Arginine is a conditionally essential amino acid in livestock and has a potential role on regulating energy partitioning between fat and lean deposition (29). Intriguingly, in lower body weight suckling lambs affected by intrauterine growth restriction, arginine supplementation increased ADG and decreased feed conversion rate (30). In the growing period in Dorper and Damara sheep, seasonal weight loss resulting from dietary restriction resulted in an increased arginine level in liver, but a decreased level in Australia Merino, while histidine level increased in all three sheep studied during dietary restriction (14). Plasma arginine was reported to increase in feed-restricted dairy cows, too (28). Collectively, these results showed that circulating amino acid concentrations changed to satisfy requirements of growth needs and normal metabolism in sheep.

Histidine can be a substrate for gluconeogenesis and protein synthesis; however, it can also affect the active site of enzymes (31). L-Histidinol and 3-Methyl-L-histidine (annotated in histidine metabolism pathway) were down-regulated in lambs with high ADG (Figure 1D and Supplementary Table 1), indicating that histidine metabolism had a trend of less activity, potentially to support faster growth and development in lambs in an intensive fattening system. Moreover, histidine metabolism seems to be up-regulated under nutritional restriction. It was reported that plasma L-histidine level decreased in barn confinement sheep compared with free grazing sheep due to high body weight gain (32).

Collectively, the purpose of this report was to reveal serum metabolome profiles of fattening lambs in a barn feeding fattening system, with particular attention to unique metabolites in lambs with high or low ADG. Our findings showed that differential metabolites affected by ADG belonged to lipids and lipid-like molecules, organic nitrogen compounds, organic acids and derivatives, benzenoids, organic oxygen compounds, and organoheterocyclic compounds. The identified metabolites have an effect on regulating metabolic pathways, biosynthesis of amino acids, glycerophospholipid metabolism, and histidine metabolism. These results indicate that selected serum metabolites could have potential application to estimate sheep with different ADG. Further larger-size studies with more various cohorts of sheep are desired to validate our finding.

## Data Availability Statement

The original contributions presented in the study are included in the article/Supplementary Material, further inquiries can be directed to the corresponding author/s.

## Ethics Statement

The animal study was reviewed and approved by Beijing Academy of Agriculture and Forestry Sciences.

## Author Contributions

TF and YL conceived the study. JW and TF obtained funding. HD performed animal trials and data collection. WX and TF performed data interpretation. TF and HD wrote the manuscript. WX and ÁK performed manuscript revision. All authors read and approved the final manuscript content.

## Conflict of Interest

The authors declare that the research was conducted in the absence of any commercial or financial relationships that could be construed as a potential conflict of interest.
