# Supplementary material for: Metabolite Profile of Sheep Serum With High or Low Average Daily Gain
Source: Front Vet Sci. 2021 May 5;8:662536. doi: 10.3389/fvets.2021.662536 (PMC8131665; doi:10.3389/fvets.2021.662536)
Supplement: Supplementary file 1 [file Table_1.DOCX]

Table 1. List of identified metabolites differentially accumulated in serum samples from high ADG lambs compared with low ADG lambs

| Metabolite (57) | VIP^1^ | *P* value | Log_2_FC^2^ | Regulation^3^ |
| --- | --- | --- | --- | --- |
| Lipids and lipid-like molecules (36) | | | | |
| Myristic acid | 1.134 | <0.001 | 0.990 | Down |
| Oleamide | 1.161 | <0.001 | 0.990 | Down |
| LysoPC(18:3(9Z,12Z,15Z)) | 1.829 | <0.001 | 1.026 | Up |
| 2-Palmitoylglycerophosphocholine | 2.697 | <0.001 | 1.050 | Up |
| PC(15:0/20:1(11Z)) | 1.789 | 0.005 | 1.025 | Up |
| PC(14:1(9Z)/P-18:1(11Z)) | 1.801 | <0.001 | 1.022 | Up |
| 1-a,24R,25-Trihydroxyvitamin D2 | 1.046 | <0.001 | 0.993 | Down |
| PE(20:5(5Z,8Z,11Z,14Z,17Z)/24:0) | 1.028 | <0.001 | 0.995 | Down |
| PE-NMe(22:6(4Z,7Z,10Z,13Z,16Z,19Z)/24:0) | 1.020 | 0.005 | 0.992 | Down |
| PC(14:1(9Z)/20:2(11Z,14Z)) | 1.446 | 0.004 | 1.015 | Up |
| PE(18:0/18:3(6Z,9Z,12Z)) | 1.308 | 0.001 | 1.012 | Up |
| PE(20:1(11Z)/P-18:1(11Z)) | 1.686 | 0.016 | 1.023 | Up |
| PC(18:3(9Z,12Z,15Z)/20:1(11Z)) | 1.554 | 0.003 | 1.017 | Up |
| PE(18:2(9Z,12Z)/P-18:0) | 1.500 | 0.007 | 1.016 | Up |
| PC(18:0/18:3(9Z,12Z,15Z)) | 1.454 | 0.002 | 1.013 | Up |
| 3,3-Dimethylglutaric acid | 1.564 | 0.022 | 1.037 | Up |
| 2-[(sulfooxy)methyl]butanoic acid | 3.098 | 0.031 | 1.156 | Up |
| D-Linalool 3-(6''-malonylglucoside) | 2.291 | 0.008 | 1.063 | Up |
| 3,4,5-trihydroxy-6-^4^ | 2.129 | 0.039 | 1.060 | Up |
| 3-Methyl-3-butenyl apiosyl-(1->6)-glucoside | 1.626 | 0.045 | 1.040 | Up |
| PE(20:0/20:4(5Z,8Z,11Z,14Z)) | 1.335 | 0.039 | 1.023 | Up |
| LysoPC(20:5(5Z,8Z,11Z,14Z,17Z)) | 2.255 | <0.001 | 1.044 | Up |
| PE-NMe2(18:1(11Z)/18:2(9Z,12Z)) | 1.657 | 0.014 | 1.029 | Up |
| PE-NMe(20:0/22:4(7Z,10Z,13Z,16Z)) | 1.927 | 0.020 | 1.040 | Up |
| PC(16:0/18:3(6Z,9Z,12Z)) | 1.414 | 0.004 | 1.018 | Up |
| PE-NMe(20:0/20:3(5Z,8Z,11Z)) | 2.063 | 0.012 | 1.045 | Up |
| PE-NMe(16:0/22:5(4Z,7Z,10Z,13Z,16Z)) | 2.186 | <0.001 | 1.036 | Up |
| PC(18:0/22:5(4Z,7Z,10Z,13Z,16Z)) | 1.599 | 0.030 | 1.026 | Up |
| PE(18:2(9Z,12Z)/20:0) | 1.445 | 0.046 | 1.023 | Up |
| LysoPC(18:3(6Z,9Z,12Z)) | 1.759 | <0.001 | 1.025 | Up |
| PE-NMe(18:0/20:1(11Z)) | 2.088 | 0.021 | 1.043 | Up |
| PE-NMe(18:1(11Z)/20:2(11Z,14Z)) | 1.151 | 0.023 | 1.013 | Up |
| PE(15:0/22:1(13Z)) | 1.410 | 0.027 | 1.018 | Up |
| PE-NMe(16:0/22:4(7Z,10Z,13Z,16Z)) | 1.587 | <0.001 | 1.017 | Up |
| PE-NMe(18:0/22:4(7Z,10Z,13Z,16Z)) | 1.581 | 0.008 | 1.022 | Up |
| PC(18:0/18:2(9Z,12Z)) | 1.351 | <0.001 | 1.017 | Up |
| Organic nitrogen compounds (5) | | | | |
| L-Histidinol | 1.715 | 0.016 | 0.974 | Down |
| N,N-Dimethylaniline | 1.266 | <0.001 | 0.987 | Down |
| L-Carnitine | 1.969 | <0.001 | 0.973 | Down |
| Isobutylpropylamine | 1.323 | <0.001 | 0.992 | Down |
| Nervonyl carnitine | 1.280 | <0.001 | 0.992 | Down |
| Organic acids and derivatives (4) | | | | |
| 3-Methyl-L-histidine | 1.612 | 0.014 | 0.979 | Down |
| L-Arginine | 1.080 | 0.026 | 0.985 | Down |
| 2-[3-(sulfooxy)phenyl]acetic acid | 1.856 | 0.046 | 1.046 | Up |
| 3-[3-(Sulfooxy)phenyl]propanoic acid | 1.973 | 0.049 | 1.053 | Up |
| Benzenoids (2) | | | | |
| D8'-Merulinic acid A | 2.633 | <0.001 | 0.949 | Down |
| 2-Dodecylbenzenesulfonic acid | 1.221 | 0.008 | 0.987 | Down |
| Organic oxygen compounds (2) | | | | |
| D-Sedoheptulose 7-phosphate | 1.306 | <0.001 | 0.987 | Down |
| Benzoquinoneacetic acid | 1.452 | <0.001 | 0.988 | Down |
| Organoheterocyclic compounds (1) | | | | |
| Bikojic acid | 1.416 | <0.001 | 0.985 | Down |
| Others (7) | | | | |
| 3-Buten-1-amine | 1.337 | 0.035 | 0.977 | Down |
| R-Beta-methylphenylethylamine | 1.191 | 0.002 | 0.986 | Down |
| (6R,8Z)-6-Hydroxy-3-oxotetradecenoic acid | 1.334 | 0.001 | 0.987 | Down |
| PC(20:5(5Z,8Z,11Z,14Z,17Z)/0:0) | 2.145 | 0.002 | 1.046 | Up |
| 4-Formylsalicylic acid | 1.607 | <0.001 | 0.984 | Down |
| Bis(2-ethylhexyl) phthalate | 1.779 | <0.001 | 0.981 | Down |
| PC(16:0/22:5(4E,7E,10E,13E,16E))[U] | 1.271 | 0.005 | 1.012 | Up |

^1^VIP = VIP_pred_OPLS-DA.

^2^FC = fold change.

^3^“up” means the serum compound is overaccumulated in high ADG lambs as compared with low ADG lambs.

^4^3,4,5-trihydroxy-6- = 3,4,5-trihydroxy-6-{[1-(4-methoxyphenyl)pentan-3-yl]oxy}oxane-2-carboxylic acid.
